# Purple perilla extracts with α-asarone enhance cholesterol efflux from oxidized LDL-exposed macrophages

**DOI:** 10.3892/ijmm.2015.2101

**Published:** 2015-02-12

**Authors:** SIN-HYE PARK, JI HUN PAEK, DAEKEUN SHIN, JAE-YONG LEE, SOON SUNG LIM, YOUNG-HEE KANG

**Affiliations:** 1 Department of Food Science and Nutrition, Hallym University, Chuncheon, Kangwon-do 200-702, Republic of Korea; 2 Department of Biochemistry, School of Medicine, Hallym University, Chuncheon, Kangwon-do 200-702, Republic of Korea

**Keywords:** α-asarone, ATP-binding cassette transporter, cholesterol efflux, oxidized low-density lipoprotein, purple perilla

## Abstract

The cellular accumulation of cholesterol is critical in the development and progression of atherosclerosis. ATP-binding cassette (ABC) transporters play an essential role in mediating the efflux of excess cholesterol. In the current study, we investigated whether purple *Perilla frutescens* extracts (PPE) at a non-toxic concentration of 1–10 *μ*g/ml stimulate the induction of the ABC transporters, ABCA1 and ABCG1, and cholesterol efflux from lipid-laden J774A.1 murine macrophages exposed to 50 ng/ml oxidized low-density lipoprotein (LDL). Purple perilla, an annual herb in the mint family and its constituents, have been reported to exhibit antioxidant and cytostatic activity, as well as to exert anti-allergic effects. Our results revealed that treatment with oxidized LDL for 24 h led to the accumulation of lipid droplets in the macrophages. PPE suppressed the oxidized LDL-induced foam cell formation by blocking the induction of scavenger receptor B1. However, PPE promoted the induction of the ABC transporters, ABCA1 and ABCG1, and subsequently accelerated cholesterol efflux from the lipid-loaded macrophages. The liver X receptor (LXR) agonist, TO-091317, and the peroxisome proliferator-activated receptor (PPAR) agonist, pioglitazone, increased ABCA1 expression and treatment with 10 *μ*g/ml PPE further enhanced this effect. PPE did not induce LXRα and PPARγ expression *per se*, but enhanced their expression in the macrophages exposed to oxidized LDL. α-asarone was isolated from PPE and characterized as a major component enhancing the induction of ABCA1 and ABCG1 in macrophages exposed to oxidized LDL. α-asarone, but not β-asarone was effective in attenuating foam cell formation and enhancing cholesterol efflux, revealing an isomeric difference in their activity. The results from the present study demonstrate that PPE promotes cholesterol efflux from macrophages by activating the interaction of PPARγ-LXRα-ABC transporters.

## Introduction

The aberrant trafficking and cellular accumulation of cholesterol are ascribed to a number of diseases, including atherosclerotic cardiovascular disease and cancer (1,2). Macrophage scavenger receptors (SRs) are membrane receptors responsible for the internalization of oxidized low-density lipoprotein (LDL) that promotes the cellular accumulation of cholesterol (3). Oxidized LDL is degraded to oxysterols that are active components present in oxidized LDL as inflammatory factors in the formation of atherosclerotic plaque (4,5). Cholesterol efflux from foam cells, the initial step of reverse cholesterol transport, is the most relevant step with respect to atherosclerosis (6). Several ATP-binding cassette (ABC) transporters are known to play a crucial role in cholesterol homeostasis by mediating cholesterol efflux and translocation (7). ABCA1 and ABCG1, membrane transporters abundant in macrophages, mediate the efflux of cholesterol and phospholipids to lipid-free apolipoproteins (apoA1 and apoE) to then form nascent high-density lipoprotein (HDL) (6,8). The disruption of ABCA1 or ABCG1 in mice has been shown to promote the accumulation of excess cholesterol in macrophages, and the physiological manipulation of ABCA1 expression affects atherogenesis (6). Thus, there are growing therapeutic approaches that target ABCA1 and ABCG1 to stimulate the flux of lipids through the reverse cholesterol transport (RCT) pathway (9).

The ligand activation of the nuclear orphan receptor, liver X receptor (LXR), by oxysterols from mitochondrial cholesterol or present in oxidized LDL is pivotal in the cellular response to an elevated sterol content, triggering cholesterol efflux mechanisms by potently upregulating the gene expression of ABCA1 and ABCG1 (6,10). The activation of the LXR pathway interferes with various mechanisms underlying the development of atherosclerotic plaque (11,12). LXR agonists have shown promise as potential therapeutics with anti-atherogenic and anti-inflammatory properties (12). Thus, the function of LXR in cholesterol efflux and inflammatory signaling make it attractive as a therapy for cardiovascular and inflammatory diseases. Peroxisome proliferator-activated receptor γ (PPARγ) is highly expressed in macrophages and foam cells of atherosclerotic lesions, and has been shown to upregulate LXR expression and elevate macrophage ABCA1 levels (8,13). Thus, the PPAR-LXR-ABCA1 interaction may be integral to cholesterol homeostasis and HDL metabolism. The therapeutic manipulation of ABC transporters is thus feasible using PPAR and LXR agonists.

Previous studies have revealed the antioxidant activities of purple perilla (*Perilla frutescens*) (14,15). In a recent study, it was demonstrated that red perilla extracts possess high antioxidant activity and prevent LDL oxidation and lipid peroxide formation *in vitro* and in human subjects (14). In another study, it was also demonstrated that an aqueous fraction of *Perilla frutescens* Britton has anti-atopic dermatitis activity in an animal model of 2,4-dinitrofluorobenzene-induced atopic dermatitis (16). It has been shown that rosmarinic acid and caffeic acid are major compounds in *Perilla frutescens* (15,17). Our nuclear magnetic resonance spectrometry study revealed that in the ethyl acetate soluble fraction of methanol extracts of purple perilla, the main compounds inhibiting aldose reductase were identified as chlorogenic acid, rosmarinic acid, luteolin and methyl rosmarinic acid (18). Rosmarinic acid in purple perilla leaves is the abundant phenolic acid with DPPH radical scavenging activity and reducing power (15). Caffeic acid from perilla leaves has also shown an additive hepatic protection with rosmarinic acid against oxidative hepatic damage (17). In addition, perilla leaf extract has been shown to ameliorate obesity and dyslipidemia induced by a high-fat diet through the downregulation of adipogenic transcription factor and specific target genes (19).

In the present study, we hyopthesized that purple perilla (hexane) extracts (PPE) may improve macrophage cholesterol efflux by activating PPARγ-LXRα-ABC transporter pathway for the clearance of excess cholesterol induced by exposure to oxidized LDL. To examine the hypothesis, we investigated whether PPE stimulates ABC transporter-mediated cholesterol efflux from lipid-laden murine macrophages. On the basis of cholesterol efflux and HDL formation, a major PPE component was identified and structurally characterized.

## Materials and methods

### Materials

Dulbecco’s modified Eagle’s medium (DMEM) chemicals, fatty acid-free bovine serum albumin (BSA), TO-091317 (LXR agonist), pioglitazone (PPAR agonist) and Oil Red O were purchased from Sigma-Aldrich Chemical Co. (St. Louis, MO, USA), as were all other reagents, unless stated otherwise. 3-(4,5-Dimethylthiazol-2-yl)-2,5-diphenyltetrazolium bromide (MTT) was obtained from Duchefa Biochemie (Haarlem, The Netherlands). Fetal bovine serum (FBS) and penicillin-streptomycin were obtained from Lonza (Basel, Switzerland). SR-B1 antibody (#sc67099) and β-asarone protein were purchased from Santa Cruz Biotechnology, Inc. (Santa Cruz, CA, USA). Antibodies to ABCA1 (#NB400-105) and ABCG1 (#NB400-132) were obtained from Novus Biologicals (Littleton, CO, USA). PPARγ antibody (#2430) was supplied by Cell Signaling Technology (Danvers, MA, USA). LXRα antibody (#PAI-332) was provided by Pierce Biotechnology (Rockford, IL, USA). α-asarone protein was purchased from Cayman Chemical Co. (Ann Arbor, MI, USA). β-actin antibody (#A5441) was purchased from Sigma Aldrich Chemical Co. Horseradish peroxidase (HRP)-conjugated goat anti-rabbit IgG (#111-035-003) was supplied from Jackson ImmunoResearch Laboratory (West Grove, PA, USA).

### Preparation and oxidation of human plasma LDL

Human plasma LDL was prepared by discontinuous density gradient ultracentrifugation as previously described (20,21). A pooled human normolipidemic plasma LDL fraction was dialyzed and used within 4 weeks. The present study for LDL isolation was approved by the Hallym University Institutional Review Board (HIRB-2011-007-2). The concentration of total cholesterol was measured using commercial diagnostic kits (Asan Pharmaceutical, Seoul, Korea). Oxidized LDL was prepared by incubation with 10 *μ*M CuSO_4_ in F-10 medium at 37°C for 24 h. The extent of LDL oxidative modification was routinely determined using thiobarbituric acid reactive substances measurements and eletrophoretic mobility assay.

### PPE preparation and identification of α-asarone

Purple perilla was purchased from a local market in Chuncheon, Korea. A voucher sample (RIC-2012-5) has been deposited at the Center for Efficacy Assessment and Development of Functional Foods and Drugs, Hallym University, Chuncheon, Korea. The specimens were authenticated by Emeritus Professor Hyung-Joon Chi, Seoul National University, Korea. Dried leaves of *Perilla frutescens* (2 kg) were extracted 3 times with 99.5% methanol for 5 h. The solvent was evaporated under reduced pressure below 45°C to yield a methanol extract (yield, 11.68%). The extract was suspended in distilled water and partitioned with n-hexane (n-Hex), methylene chloride (CH_2_Cl_3_), ethyl acetate (EtOAc), n-butanol (n-BuOH), and H_2_O to yield n-Hex (40.83 g), EtOAc (25.20 g), CH_2_Cl_3_ (22.24 g), n-BuOH (116.88 g) and H_2_O fractions (27.42 g). A portion of the n-Hex fraction (PPE) was purified by chromatography on a silica gel eluted with chloroform and increasing proportion methanol (10:0-9:1) to yield 11 parts (parts 1–11). The ability of each fraction to reduce the viability of DU145 human prostate cancer cells was evaluated by MTT assay. The part 5 (0.46 g) revealing the most potent activity was further purified by recrystallization to yield compound 1 (76 mg).

The ^1^H- and ^13^C-nuclear magnetic resonance (NMR) spectra of the isolated pure compound 1 were recorded with a Bruker DPX 400 NMR spectrometer (400 MHz), using DMSO-*d*_6_ as a solvent. Compound 1: ^1^H-NMR (CD_3_OD, 400 MHz) δ 1.82 (3H, dd, *J*=6.5, 1.6 Hz, H-3′), δ 3.17 (3H, s, -OCH_3_), δ 3.76 (3H, s, -OCH_3_), δ 3.78 (3H, s, -OCH_3_), δ 6.11 (1H, dq, *J*=10.6, 6.6 Hz, H-2′), δ 6.56 (1H, dd, *J*=16, 1.6 Hz, H-1′), δ 6.63 (1H, s, H-3), δ 7.25 (1H, s, H-6); ^13^C-NMR (CD_3_OD, 100 MHz) δ 19.42 (C-3′), 56.56 (-OCH_3_), 57.02 (-OCH_3_), 57.09 (-OCH_3_), 99.22 (C-3), 110.91 (C-6), 118.43 (C-5), 124.12 (C-2′), 125.79 (C-1′), 143.81 (C-1), 149.65 (C-2), 151.16 (C-4); ESI-MS (m/z) 209 [M+H]^+^; 193.1 [M-CH3]^+^, 165.1 [M-CH3CO]^+^; UV (MeCN, λ_max_ nm) 212, 258, 312. The ^1^H-NMR, ^13^C-NMR, ESI-MS and UV data confirmed compound 1 as α-asarone, according to the isolation and identification described in a previous study (22).

### Cell culture and Oil Red O staining

**Figure 1:**
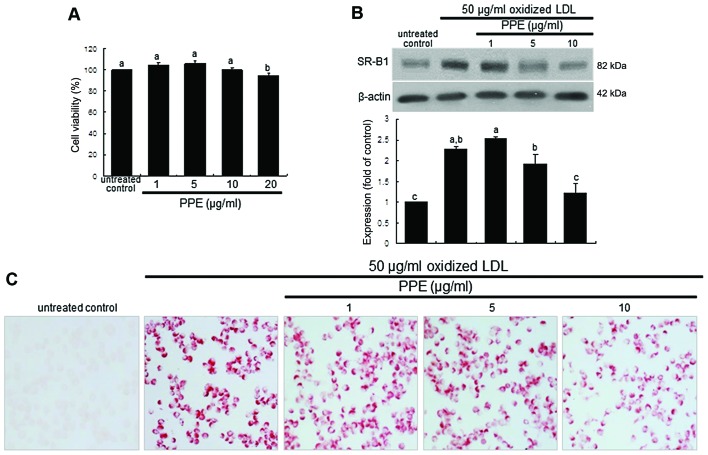
Effects of purple *Perilla frutescens* extracts (PPE) on foam cell formation. (A) Cytotoxicity of PPE; (B) inhibition of scavenger receptor (SR)-B1 induction; and (C) intracellular lipid accumulation following treatment with PPE in 50 *μg*/ml Cu^2+^-oxidized low-density lipoprotein (LDL)-exposed J774A.1 murine macrophages. (A) MTT assay was performed for the measurement of PPE toxicity. Graph data represent 1 of 4 independent experiments with multiple estimations. Values are expressed as the percentage of cell survival relative to the untreated control cells (cell viability, 100%). (B) For the measurement of SR-B1 expression, total cell lysates were subjected to western blot analysis with a primary antibody against SR-B1. β-actin was used as an internal control. Bar graphs (n=3) represent quantitative densitometric results of the upper bands. (C) Foam cell formation was measured by staining the macrophages with Oil Red O. Microphotographs were obtained using an optical microscope. Magnification, x200. Bar graphs (means ± SEM) denoted without a common letter indicate significant difference, P<0.05.

The mouse macrophage-like cell line, J774A.1 (#TIB-67; American Type Culture Collection, Manassas, VA, USA), was grown in DMEM supplemented with 10% FBS at 37°C in a humidified atmosphere of 5% CO_2_ in air. The viability of the J774A.1 cells was determined using a colorimetric assay based on the uptake of MTT by viable cells. There was no noticeable cytotoxicity observed with the dose of ≤10 *μ*g/ml PPE (Fig. 1A). The macrophages were pre-treated with 1–10 *μ*g/ml PPE and exposed to 50 *μ*g/ml oxidized LDL or the other agonists, 10 *μ*M pioglitazone or 1 *μ*M TO-091317, for various periods of time. The J774A.1 macrophages were incubated in DMEM supplemented with 0.4% fatty acid-free BSA and treated with CuSO_4_-oxidized LDL.

Oil Red O staining was used to visualize the lipid uptake and foam cell formation in the macrophages. Oil Red O is a fat-soluble dye used for staining lipids and some lipoproteins. Following the culture of J774A.1 cells with 50 *μ*g/ml oxidized LDL in the absence or presence of PPE, the cells were treated with 0.5% Oil Red O dissolved in 60% 2-propanol. After mounting, images were obtained using an optical microscope (AXIO Imager microscope; Carl Zeiss, Oberkochen, Germany).

### Western blot analysis

Following the culture protocols, the cells were lysed in lysis buffer. Equal protein amounts of cell lysates were electrophoresed on a 6–8% sodium dodecyl sulfate-polyacrylamide gel (SDS-PAGE) and transferred onto a nitrocellulose membrane. After blocking with 5% skim milk, the membrane was incubated with polyclonal rabbit antibodies to SR-B1, ABCA1, ABCG1, PPARγ and LXRα. Following triple washes, the membrane was incubated for 1 h with a goat anti-rabbit IgG conjugated to HRP. The individual protein level was determined using Immobilon Western Chemiluminescnet HRP substrate (Millipore, Billerica, MA, USA). Incubation with mouse β-actin antibody was also performed for comparative controls. After performing immunoblotting, the blot bands were visualized on Agfa X-ray film (Agfa-Gevaert, Mortsel, Belgium).

### Cholesterol efflux assay

The J774A.1 macrophages were treated with 1–10 *μ*g/ml PPE or 1–10 *μ*M α-asarone for 12 h and then equilibrated with 1 *μ*g/ml 3-dodecanoyl-NBD-labeled cholesterol (Cayman Chemical Co.) for an additional 6 h. The cells exposed to NBD-labeled cholesterol were washed with phosphate-buffered saline for various periods of time and freshly incubated in DMEM for 6 h. Fluorescence-labeled cholesterol released from the cells into the medium for 6 h was detected using a fluorometer at ranges from λ=485 to 538 nm (Fluoroskan Ascent FL, #5210450; Thermo Scientific, Waltham, MA, USA). Cholesterol efflux was expressed as the percentage fluorescence in the medium relative to the total fluorescence.

### RT-PCR

Following the culture protocols, total RNA was isolated from the J774A.1 macrophages using a commercially available TRIzol reagent kit (Molecular Research Center, Cincinnati, OH, USA). RNA (5 *μ*g) was reverse transcribed with 200 units of reverse transcriptase (Promega Corp., Madison, WI, USA) and 0.5 mg/ml oligo(dT)_15_ primer (Bioneer, Daejeon, Korea). RT-PCR was also performed for semi-quantifying the levels of the mRNA transcripts of SR-B1 and retinoid X receptor (RXR)α. The PCR conditions for SR-B1 [5′-ATGGGCCAGCGTGCTTTTATGA-3′ (forward) and 5′-AACCACAGCAACGGCAGAACTA-3′ (reverse) 752 bp] were 94°C (3 min), and 30 cycles at 94°C (30 sec), 60°C (45 sec) and 72°C (45 sec) and the conditions for RXRα [5′-CAATGGCGTCCTCAAGGTTC-3′ (forward) and 5′-ACTCCACCTCGTTCTCATTC-3′ (reverse) 326 bp)] were 95°C (10 min) and 35 cycles at 94°C (30 sec), 60°C (45 sec), and 72°C (45 sec). The housekeeping gene, glyceraldehyde 3-phosphate dehydrogenase (GAPDH) [5′-AACTTTGGCATTGTGGAAGGG-3′ (forward) and 5′-GACACATTGGGGGTAGGAACAC-3′ (reverse) 224 bp], was used for internal normalization for the co-amplification with the respective gene.

### Data analysis

The results are presented as the means ± SEM. Statistical analyses were conducted using the SAS software package version 6.12 (SAS Institute, Cary, NC, USA). One-way ANOVA was used to determine the inhibitory effects of PPE on the effects of oxidized LDL on macrophages. Differences between the treatment groups were analyzed using Duncan’s multiple-range test and considered statistically significant at a value of P<0.05.

## Results

### Suppression of SR-B1 induction and foam cell formation by PPE

The internal uptake of oxidized LDL is known to require SR induction in macrophages (23). Oxidized LDL rapidly increased SR-B1 expression within 2 h, which was sustained for up to 8 h (data not shown). When the cells were exposed to oxidized LDL for 6 h and treated with 1–10 *μ*g/ml PPE, SR-B1 expression decreased in a dose-dependent manner (Fig. 1B). A further experiment was conducted to examine intracellular lipid accumulation in macrophages, evidenced by using Oil Red O staining. There was strong reddish staining observed in the macrophages exposed to 50 *μ*g/ml oxidized LDL for 18 h. This indicates the cellular accumulation of lipids through the upregulation of SR-B1. When the macrophages were treated with 10 *μ*g/ml PPE for 18 h, the number of reddish lipid droplets decreased (Fig. 1C). Thus, PPE delayed foam cell formation in the macrophages exposed to 50 *μ*g/ml oxidized LDL.

### Increase in ABCA1 and ABCG1 expression and cholesterol efflux by PPE

**Figure 2:**
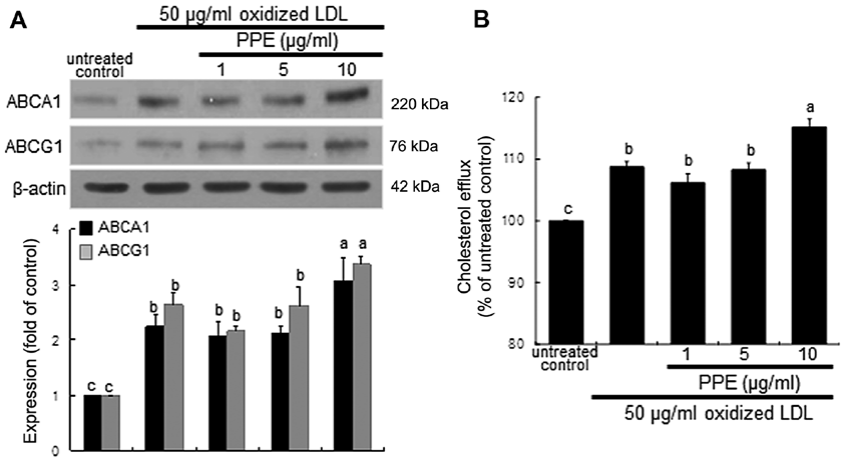
(A) Upregulation of ABCA1 and ABCG1 and (B) enhancement of cholesterol efflux by purple *Perilla frutescens* extracts (PPE) in 50 *μ*g/ml Cu^2+^-oxidized low-density lipoproteins (LDL)-exposed J774A.1 murine macrophages. (A) For the measurement of ABCA1 and ABCG1 expression, total cell lysates were subjected to western blot analysis with a primary antibody against ABCA1 or ABCG1. β-actin was used as an internal control. Bar graphs (means ± SEM, n=3) represent quantitative densitometric results of the upper bands. (B) Cholesterol efflux was expressed as the percentage of fluorescence in the medium relative to the total fluorescence. Bar graphs denoted without a common letter indicate significant difference, P<0.05.

The membrane proteins, ABCA1 and ABCG1, play a role in cholesterol efflux from lipid-laden macrophages (6,8). Exposure of the macrophages for 8 h to oxidized LDL highly promoted the induction of ABCA1 and ABCG1. When 10 *μ*g/ml PPE was applied to the oxidized LDL-exposed macrophages, the expression of these proteins was further increased (Fig. 2A). Thus, PPE promotes cholesterol efflux from lipid-laden foam cells. As expected, the cholesterol efflux was enhanced in the macrophages exposed to 50 *μ*g/ml oxidized LDL and was further accelerated by treatment with 10 *μ*g/ml PPE (Fig. 2B).

### Promoting effect of PPE on ABCA1 expression induced by LXR

**Figure 3:**
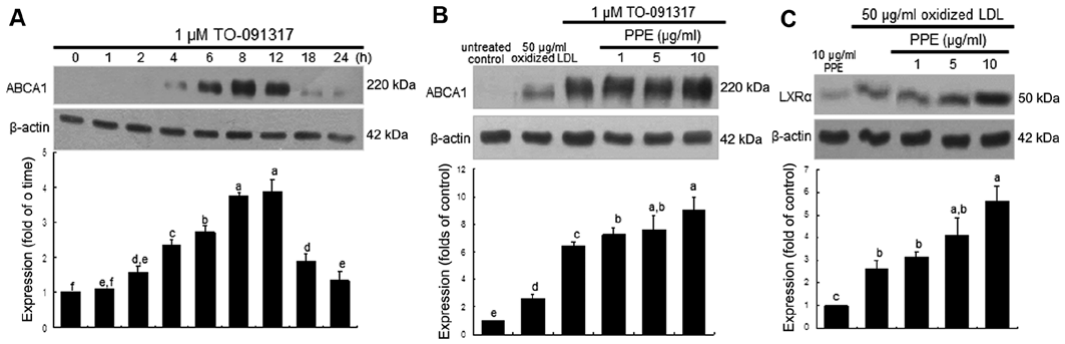
(A) Time course response of ABCA1 induction by TO-091317 and (B) upregulation of ABCA1 by purple *Perilla frutescens* extracts (PPE), and (C) enhancement of liver X receptor (LXR)α induction by PPE. J774A.1 murine macrophages were cultured with 1 *μ*M TO-091317 or 50 *μ*g/ml Cu^2+^-oxidized low-density lipoprotein (LDL) in the absence or presence of 1–10 *μ*g/ml PPE. For the measurement of expression of (A and B) ABCA1 and (C) LXRα, total cell lysates were subjected to western blot analysis with a primary antibody against ABCA1 or LXRα. β-actin was used as an internal control. Bar graphs (means ± SEM, n=3) represent quantitative densitometric results of the upper bands. Bar graphs denoted without a common letter indicate significant difference, P<0.05.

We wished to confirm whether the upregulation of ABCA1 entails the nuclear induction of LXR, which was further accelerated by the administration of PPE. ABCA1 expression was highly induced within 4 h in the macrophages stimulated with the LXR agonist, TO-091317, at 1 *μ*M and was sustained at high levels for up to 12 h (Fig. 3A). It should be noted that the induction of ABCA1 by TO-091317 was much more prominent than that induced by oxidized LDL (Fig. 3B). When the J774A.1 macrophages were treated with 1 *μ*M TO-091317 for 8 h, the induction of ABCA1 was accelerated by treatment with 10 *μ*g/ml PPE (Fig. 3B). In addition, the induction of LXRα by oxidized LDL was further enhanced in the PPE-treated macrophages (Fig. 3C). However, PPE *per se* did not induce nuclear LXRα expression (Fig. 3C). This indicated that the nuclear induction of LXRα by PPE in the presence of oxidized LDL appeared to be synergistic to that of TO-091317, which led to an increase in ABCA1 expression and cholesterol efflux.

### Increase in PPARγ expression by PPE

**Figure 4:**
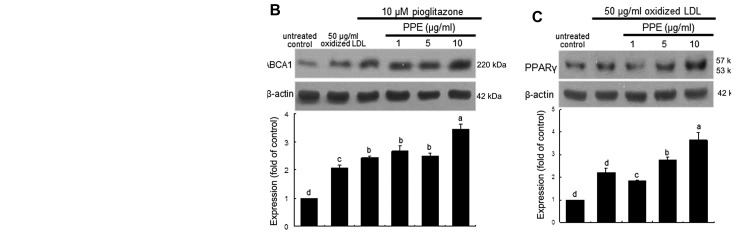
(A) Time course response of ABCA1 induction by pioglitazone, and (B) enhancement of ABCA1 and (C) peroxisome proliferator-activated receptor γ (PPARγ) by purple *Perilla frutescens* extracts (PPE). J774A.1 murine macrophages were incubated with 10 *μ*M pioglitazone and 50 *μ*g/ml oxidized low-density lipoprotein (LDL) in the absence or presence of 1–10 *μ*g/ml PPE. For the measurement of expression of (A and B) ABCA1 and (C) PPARγ, total cell lysates were subjected to western blot analysis with a primary antibody against ABCA1 or PPARγ. β-actin was used as an internal control. Bar graphs (means ± SEM, n=3) represent quantitative densitometric results of the upper bands. Bar graphs denoted without a common letter indicate significant difference, P<0.05.

PPARγ is highly expressed in macrophages and has been shown to elevate ABCA1 levels (8,13). As expected, ABCA1 expression was temporally induced from 6 h in the J774A.1 macrophages stimulated with the PPARγ agonist, pioglitazone at 10 *μ*M, and was sustained at high levels for up to 24 h (Fig. 4A). Treatment with PPE at 10 *μ*g/ml further increased ABCA1 expression in the macrophages following 18 h of treatment with 10 *μ*M pioglitazone (Fig. 4B). The induction of PPARγ was promoted to an even greater extent in the oxidized LDL-treated macrophages, and this increase was even further elevated by PPE (Fig. 4C). These results suggest that PPE promotes the activation of the PPAR-LXR-ABCA1 pathway, leading to an increase in cholesterol efflux.

### Induction of ABCA1 by PPE-α-asarone

**Figure 5:**
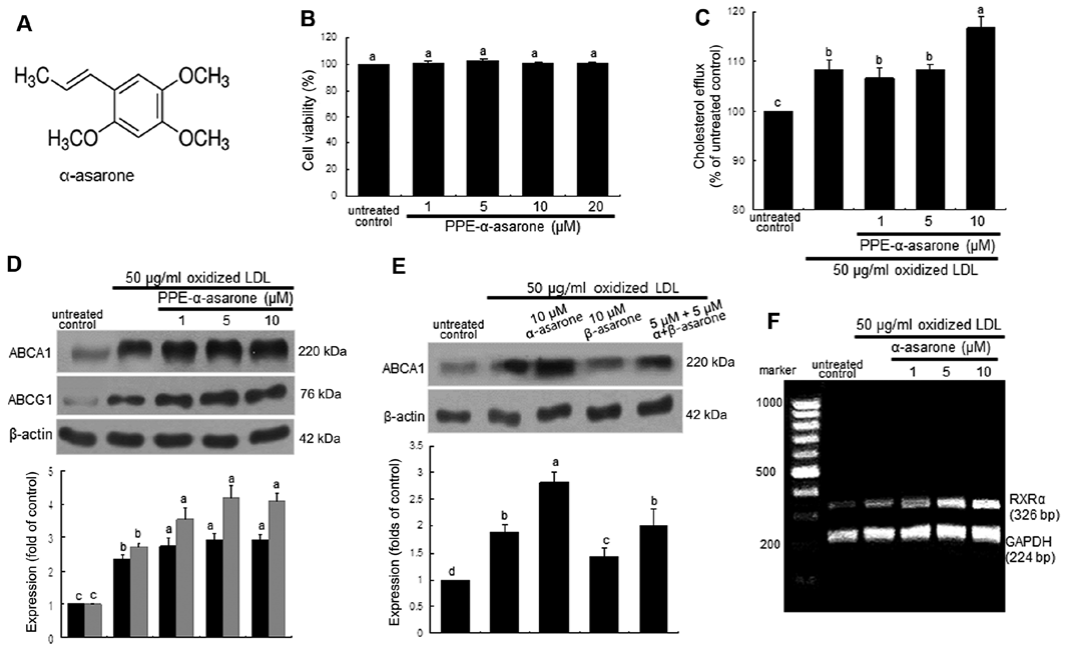
(A) Chemical structure, (B) cytotoxicity of α-asarone, (C) enhancement of cholesterol efflux by α-asarone, (D and E) upregulation of ABCA1 and ABCG1 by α-asarone and β-asarone, and (F) elevation of retinoid X receptor (RXR)α transcription. J774A.1 murine macrophages were exposed to 50 *μ*g/ml oxidized low-density lipoprotein (LDL) and treated with 1–10 *μ*M purple *Perilla frutescens* extracts (PPE)-α-asarone and 5–10 *μ*M β-asarone. (B) MTT assay was performed for the measurement of α-asarone toxicity. Graph data represent 1 of 4 independent experiments with multiple estimations. Values are expressed as the percentage cell survival relative to the untreated control cells (cell viability, 100%). (C) Cholesterol efflux was expressed as the percentage fluorescence in the medium relative to total fluorescence. (D and E) For the measurement of ABCA1 and ABCG1 expression, total cell lysates were subjected to western blot analysis with a primary antibody against ABCG1 or ABCG1. β-actin was used as an internal control. Bar graphs (means ± SEM, n=3) represent quantitative densitometric results of the upper bands. Bar graphs denoted without a common letter indicate significant difference, P<0.05. (F) RXRα mRNA expression was measured by RT-PCR. GAPDH was used as a housekeeping gene for the co-amplification with RXRα.

α-asarone (Fig. 5A) (PPE-α-asarone), which was isolated and identified as the major effective component in PPE, did not exert any toxic effects on the macrophages (Fig. 5B). The cholesterol efflux enhanced by oxidized LDL was further enhanced in the macrophages treated with 10 *μ*M α-asarone (Fig. 5C). Consistently, the induction of ABCA1 and ABCG1 by treatment with 50 *μ*g/ml oxidized LDL for 8 h was further upregulated by treatment with ≥1 *μ*M α-asarone isolated from PPE (Fig. 5D). By contrast, treatment with 10 *μ*M β-asarone did not exert such an induction in the oxidized LDL-exposed macrophages (Fig. 5E). Thus, the promotion of cholesterol efflux from lipid-laden foam cells by PPE may be due to the mechanistic action of α-asarone present in PPE. On the other hand, the ligand activation of RXRα was achieved by exposure of the macrophages to oxidized LDL and this effect was further enhanced by treatment with ≥5 *μ*M α-asarone (Fig. 5F).

### Inhibition of lipid uptake by PPE-α-asarone

**Figure 6:**
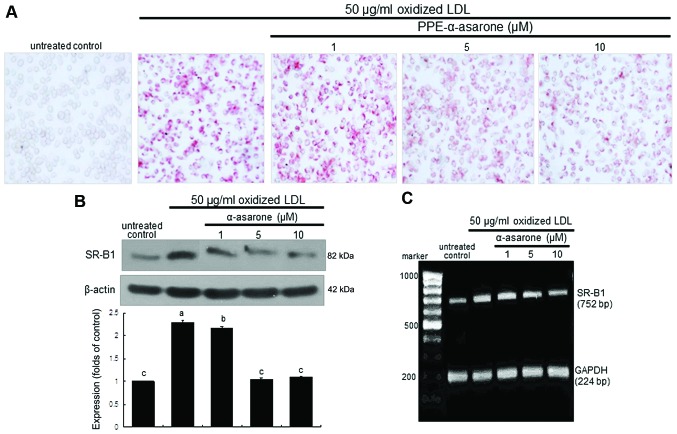
(A) Inhibition of intracellular lipid accumulation, (B) attenuation of scavenger receptor (SR)-B1 protein induction and (C) its transcription in 1–10 *μ*M purple *Perilla frutescens* extracts (PPE)-α-asarone-treated and 50 *μ*g/ml Cu^2+^-oxidized low-density lipoproteins (LDL)-exposed J774A.1 murine macrophages. (A) Foam cell formation was measured by staining the macrophages with Oil Red O. Microphotographs were obtained using an optical microscope. Magnification, x200. (B) For the measurement of SR-B1 expression, total cell lysates were subjected to western blot analysis with a primary antibody against SR-B1. β-actin was used as an internal control. Bar graphs (n=3) represent quantitative densitometric results of the upper bands. Bar graphs (means ± SEM) denoted without a common letter indicate significant difference, P<0.05. (C) SR-B1 mRNA expression was measured by RT-PCR. GAPDH was used as a housekeeping gene for the co-amplification with SR-B1.

The intracellular lipid accumulation was diminished in the macrophages exposed to 50 *μ*g/ml oxidized LDL and treated with 10 *μ*M PPE-α-asarone (Fig. 6A). The expression of SR-B1 induced by oxidized LDL was decreased in a dose-dependent manner by treatment with 1–10 *μ*M α-asarone isolated from PPE (Fig. 6B). Treatment with non-toxic α-asarone at 10 *μ*M diminished SR-B1 transcription, indicating that the induction of SR-B1 by LDL was disrupted at the transcriptional level (Fig. 6C). Thus, α-asarone from PPE is effective in blocking foam cell formation in macrophages.

## Discussion

Seven major findings were observed in this study. i) non-cytotoxic PPE at ≤10 *μ*g/ml attenuated oxidized LDL uptake and cellular lipid accumulation in J774A.1 murine macrophages by reducing the induction of SR-B1; ii) treatment of the macrophages with 10 *μ*g/ml PPE enhanced the oxidized LDL-induced expression of ABCA1 and ABCG1 and subsequent cholesterol efflux; iii) ABCA1 expression was highly upregulated in the macrophages stimulated with the LXR agonist, TO-091317, and these effects were further enhanced by treatment with 10 *μ*g/ml PPE; iv) the induction of LXRα by oxidized LDL was elevated in a dose-dependent manner by PPE; v) ABCA1 expression was further enhanced by PPE in the macrophages exposed to the PPAR agonist, pioglitazone, and treatment with 10 *μ*g/ml PPE further enhanced the expression of PPAR induced by oxidized LDL; vi) α-asarone was identified as a major compound present in the hexane extract, PPE, enhancing cholesterol efflux from lipid-laden foam cells; and vii) non-toxic α-asarone stimulated the protein expression of ABCA1 and ABCG1 concomitant with the ligand activation of RXRα, whereas SR-B1 induction and foam cell formation were blocked by this compound at the transcriptional level. These observations demonstrate that non-cytotoxic α-asarone (in PPE) is effective in blocking oxidized LDL internalization by disrupting the induction of SR-B1 and promoting the ABCA1-mediated cholesterol efflux from lipid-laden foam cells by triggering PPARγ-LXRα signaling.

Macrophage membrane SR is responsible for the uptake of oxidized LDL and promotes the cellular accumulation of cholesterol (3). The aberrant cellular accumulation of cholesterol has been implicated in the development of atherosclerosis (23,24). In the present study, we demonstrated that macrophages became lipid droplet-loaded foam cells due to oxidized LDL. Any surplus of cholesterol is released from the cell through ABC transporters for the regulation of cellular cholesterol homeostasis by mediating cholesterol efflux (1,7). As expected, oxidized LDL induced the expression of the atheroprotective membrane proteins, ABCA1 and ABCG1, and enhanced cholesterol efflux from lipid-laden foam cells. Ongoing research using animals and cells has produced increasing evidence that cholesterol efflux pathways are mediated by ABC transporters and that HDL suppresses atherosclerosis (25). In addition, the specific knockout of macrophage transporters has confirmed their role in the suppression of inflammatory responses in the arterial wall (26,27). Thus, the activation of cholesterol efflux pathways targeting ABCA1 and ABCG1 may prove to be novel therapeutic approaches to the treatment of atherosclerosis (9).

Phenolic compounds with high antioxidant activity, such as rosmarinic acid and caffeic acid have been characterized in purple perilla leaves (15,17,18). Perilla leaf extract ameliorates high-fat diet-induced obesity and dyslipidemia by downregulating epididymal adipose tissue genes, including CoA carboxylase and PPARγ (19). In addition, ethyl acetate extracts of purple perilla have been shown to inhibit the activity of aldose reductase, which is involved in diabetic complications (18). In this study, we found that PPE deterred SR-B1-mediated cholesterol internalization leading to foam cell formation, and enhanced ABC transporter-mediated cholesterol efflux from lipid-laden murine macrophages. This study attempted to characterize the major components of PPE which promote cholesterol efflux from macrophages. The active hexane fractions from PPE were isolated by conducting bioactivity-guided fractionation. Based on NMR and mass spectrometric analyses, the active ingredient responsible for the induction of ABCA1 was identified as α-asarone (2,4,5-trimethoxyphenyl-2-propene). α-asarone and β-asarone may be potential therapeutic agents for managing cognitive impairment due to their neuroprotective and cognitive-enhancing properties (28,29). α-asarone has been shown to inhibit HMG-CoA reductase, lower serum LDL cholesterol levels and to reduce the biliary cholesterol saturation index in hypercholesterolemic rats (30). In the present study, non-toxic α-asarone enhanced the expression of ABCA1 and ABCG1 induced by oxidized LDL and activated the cholesterol efflux pathway-mediated HDL formation. However, β-asarone did not show such atheroprotective effects related to cholesterol efflux. Thus, there was an isomeric difference in the induction of the cholesterol efflux-associated membrane proteins, ABCA1 and ABCG1.

The ligand activation of LXR by oxysterols from mitochondrial cholesterol or present in oxidized LDL initiates the cholesterol efflux pathway by enhancing ABCA1 and ABCG1 gene expression (6,10). Since the activation of the LXR pathway attenuates atherosclerotic plaque development, LXR agonists have shown promise as potential therapeutics (11). This study demonstrated that the LXR agonist, TO-091317, elevated ABCA1 expression in murine macrophages exposed to oxidized LDL, which was further enhanced by the presence of PPE. Thus, it can be hypothesized that PPE is an LXR agonist. Since PPE *per se* did not elicit the induction of LXRα, PPE appeared to be synergistic to TO-091317 in inducing ABCA1 expression. Furthermore, PPAR agonists, such as glitazones may be other therapeutic manipulators of ABC transporters. PPARγ is known to upregulate LXR expression and elevate macrophage ABCA1 levels (8,13). In this study, PPE enhanced the expression of PPARγ induced in the macrophages by oxidized LDL, indicating the activation of the PPARγ-LXRα pathway to promote ABCA1 protein expression. α-asarone also promoted the oxidized LDL-induced ligand activation of the nuclear receptor RXRα dimerized with LXR in facilitating the binding and transactivation of receptors with DNA response elements. Gemfibrozil analogues conjugated with α-asarone have been shown to activate the PPARα receptor and enhance the activity of the PPARα-regulated carnitine palmitoyltransferase IA gene involved in fatty acid catabolism (31). Thus, α-asarone may activate the PPARγ-LXRα-RXRα pathway for the induction of ABC transporters. On the other hand, ABCA1 protein stabilization by PPE or α-asarone may be another therapeutic mechanism. ABCA1 protein degradation may be promoted in atherosclerotic plaque milieu, thus compromising cholesterol efflux. Although the present findings are promising, further studies are required to elucidate the efficacy and side-effects of PPE and α-asarone for future use in human subjects.

In conclusion, in the current study, we demonstrated that α-asarone-rich PPE encumbered oxidized LDL uptake and cholesterol influx in J774A.1 murine macrophages by reducing the early expression of SR-B1. In addition, PPE promoted cholesterol efflux from lipid-loaded macrophages by inducing the expression of ABCA1 and ABCG1. The induction of ABCA1 by PPE was mediated through the activation of the PPARγ-LXRα-responsive cellular signaling pathway, thus transferring effluxed cholesterol onto lipid-poor apolipoproteins, thus promoting the formation of HDL particles. Thus, PPE functions as an agonist of LXR and PPARγ in macrophages, and α-asarone, which is present in PPE may be effective in cellular cholesterol handling in lipid-loaded macrophages. Although PPE may serve as a beneficial modulator against cholesterol efflux-associated atherogenesis *in vitro*, its role *in vivo* remains to be elucidated.
